# Low Expression of DYRK2 (Dual Specificity Tyrosine Phosphorylation Regulated Kinase 2) Correlates with Poor Prognosis in Colorectal Cancer

**DOI:** 10.1371/journal.pone.0159954

**Published:** 2016-08-17

**Authors:** Haiyan Yan, Kaishun Hu, Wenjing Wu, Yu Li, Huan Tian, Zhonghua Chu, H. Phillip Koeffler, Dong Yin

**Affiliations:** 1 Guangdong Provincial Key Laboratory of Malignant Tumor Epigenetics and Gene Regulation, Medical Research Center, Sun Yat-Sen Memorial Hospital, Sun Yat-Sen University, Guangzhou, 510120, China; 2 Department of Clinical Laboratory, Sun Yat-Sen Memorial Hospital, Sun Yat-Sen University, Guangzhou, 510120, China; 3 Department of Breast Oncology, Sun Yat-Sen Memorial Hospital, Sun Yat-Sen University, Guangzhou, 510120, China; 4 Department of Breast Oncology, Guangdong Hospital of Traditional Chinese Medicine, Traditional Chinese Medicine University of Guangzhou, Guangzhou, 510120, China; 5 Department of Gastrointestinal Surgery, Sun Yat-Sen Memorial Hospital, Sun Yat-Sen University, Guangzhou, China; 6 Division of Hematology/Oncology, Cedars-Sinai Medical Center, University of California Los Angeles (UCLA) School of Medicine, Los Angeles, California, United States of America; 7 National University of Singapore (CSI, NCIS), Singapore, Singapore; Sapporo Ika Daigaku, JAPAN

## Abstract

Dual-specificity tyrosine-phosphorylation-regulated kinase 2 (DYRK2) is a member of dual-specificity kinase family, which could phosphorylate both Ser/Thr and Tyr substrates. The role of DYRK2 in human cancer remains controversial. For example, overexpression of DYRK2 predicts a better survival in human non-small cell lung cancer. In contrast, amplification of DYRK2 gene occurs in esophageal/lung adenocarcinoma, implying the role of DYRK2 as a potential oncogene. However, its clinical role in colorectal cancer (CRC) has not been explored. In this study, we analyzed the expression of DYRK2 from Oncomine database and found that DYRK2 level is lower in primary or metastatic CRC compared to adjacent normal colon tissue or non-metastatic CRC, respectively, in 6 colorectal carcinoma data sets. The correlation between DYRK2 expression and clinical outcome in 181 CRC patients was also investigated by real-time PCR and IHC. DYRK2 expression was significantly down-regulated in colorectal cancer tissues compared with adjacent non-tumorous tissues. Functional studies confirmed that DYRK2 inhibited cell invasion and migration in both HCT116 and SW480 cells and functioned as a tumor suppressor in CRC cells. Furthermore, the lower DYRK2 levels were correlated with tumor sites (P = 0.023), advanced clinical stages (P = 0.006) and shorter survival in the advanced clinical stages. Univariate and multivariate analyses indicated that DYRK2 expression was an independent prognostic factor (P < 0.001). Taking all, we concluded that DYRK2 a novel prognostic biomarker of human colorectal cancer.

## Introduction

Dual-specificity tyrosine-phosphorylation-regulated kinase, including dual-specificity yak-related kinases (DYRKs) represent a large family of dual-specificity kinase, which can phosphorylate both Ser/Thr and Tyr substrates. To date, at least seven DYRK family members have been identified (*DYRK1A*, *DYRK1B*, *DYRK1C*, *DYRK2*, *DYRK3*, *DYRK4A* and *DYRK4B)* [1]. The DYRKs are evolutionarily conserved in their kinase domain, but differ from each other in the N- and C-terminal regions. These kinase domains have several distinct amino-acid sequences, including a DYRK homology (DH) box immediately preceding the kinase domain, a YXY motif in the kinase-domain-activation loop between subdomains VII and VIII, an SSC motif following sub domain VII, and conserved sequences HCDLKPEN and YXYIQSRFYR (S/A) PE in subdomains VI and VIII [2].

Emerging evidences indicated that DYRKs are pleiotropic regulators of widespread cellular functions including cell survival, cell differentiation, gene transcription and endocytosis [3]. DYRK1A/1B and DYRK3 may serve as protective kinases against apoptosis [4–6]. In specific tissues, such as neurons, muscle and blood cells, DYRK1A/1B and DYRK3 also play a role during cell differentiation [7–9]. Conversely, DYRK2 behaves as a proapoptotic kinase through phosphorylation of p53 at S46 which promotes its apoptotic activity [10]. Down-regulation of DYRK2 could also induce a G2/M arrest [11].

Abundant studies have demonstrated that DYRKs are related to human diseases. For example, increased expression of DYRK1A occurred in Down syndrome [12], as well as different types of solid tumors [13]. Interestingly, the role of DYRK2 in human cancer remains controversial. Yamashita *et al* reported that DYRK2 overexpression predicted a better survival in human non-small cell lung cancer, implying a tumor suppressor role [14]. Likewise, higher DYRK2 expression in breast cancer and pulmonary adenocarcinoma may enhance survival [15, 16]. And yet others have noted amplification and overexpression of DYRK2 in esophageal and lung adenocarcinoma, as well as gastric stromal tumor, suggesting that DYRK2 can behave as a potential oncogene [17–19], although the protein levels of the gene were not examined.

In this study, we investigated the prognostic significance of DYRK2 expression of in 181 archived CRC samples. Our study offers the first piece of evidence that shows the significant correlation between lower protein level of DYRK2 and tumor location in rectum, advanced tumor stage and unfavorable prognosis in clinical stage III and IV CRC patients. This study also confirmed that decreased expression of DYRK2 happened in rectal cancer more often than in colon cancer.

## Methods

### Ethics

The use of tissues for this study has been approved by the Ethics Committee of Sun Yat-Sen Memorial Hospital, Sun Yat-Sen University. At the time of initial diagnosis, all patients had provided written consent in the sense that their tumor samples could be used for the investigation's purpose.

### Oncomine databases

The Oncomine Platform, from web applications to translational bioinformatics services, provides solutions for individual researchers and multinational companies, with robust, peer-reviewed analysis methods and a powerful set of analysis functions that compute gene expression signatures, clusters, and gene-set modules, automatically extracting biological insights from the data. At present, the Oncomine has been contained by about 48 million gene expression measurements 65 sets of gene expression data set for use by researchers [20]. The detail statements for 6 colorectal carcinoma data sets information have been listed in the Table 1.

**Table 1:** The detail information of the six Oncomine datasets.

Dataset Name	Study Description	Array Types	Experiment Type
Gaedcke Colorectal Dataset	Sixty-five (65) rectal adenocarcinomas and their paired normal rectal mucosa samples were analyzed. [20]	Agilent Human Genome 44K,Measured 19,189 genes, 41,000 reporters.	mRNA
Skrzypczak Colorectal 2 Dataset	Forty (40) microdissected samples (5 replicates each of 8 types of epithelial or mucosa cells from tumor or normal tissues) were analyzed. [21]	Human Genome U133 Plus 2.0 Array, Measured 19,574 genes, 54,675 reporters.	mRNA
Kaiser Colon Dataset	One hundred (100) colorectal carcinoma and 5 normal colon samples were analyzed. [22]	Human Genome U133 Plus 2.0 Array, Measured 19,574 genes, 54,675 reporters.	mRNA
TCGA Colorectal Dataset	One hundred forty-six (146) colon adenocarcinoma, 69 rectal adenocarcinoma, 19 paired normal colon, and 3 paired normal rectum tissue samples were analyzed. [23]	Platform not pre-defined in Oncomine, Measured 20,423 genes, 111,123 reporters.	mRNA
Bittner Colon Dataset	Three-hundred seventy-three (373) colorectal carcinoma samples were analyzed.	Human Genome U133 Plus 2.0 Array, Measured 19,574 genes, 54,675 reporters.	mRNA
Ki Colon Dataset	One hundred and three (103) matched samples from 27 colorectal cancer patients were analyzed. [24]	Platform not pre-defined in Oncomine, Measured 9,256 genes, 15,783 reporters.	mRNA

### RNA extraction, reverse transcription and real-time PCR

Total RNAs from 16 pairs of tumor tissues and non-tumorous tissues were extracted by using the miRNeasy Mini Kit (Qiagen #217004) according to the manufacturer’s instruction. The clinicopathologic information of the 16 CRC samples was described in Table 2. Real-time PCR was performed according to standard methods as described previously [25]. GAPDH was used as an internal control. Sequences of the primers for qPCR were listed as follows, DYRK2 sense: 5’ CCTGAACAAGCAATGAAGCA; DYRK2 antisense: 5’ GGTCATCATCATAGCCACCA; GAPDH sense: 5’AGAAGGCTGGGGCTCATTTG; GAPDH antisense: 5’ GACAAGCTTCCCGTTCTCAG.

**Table 2:** The clinicopathologic features of the 16 CRC samples.

	Number of cases (%)
**Gender**	
Male	10 (62.5)
Female	6 (37.5)
**Age (years)**	
≤ 50	4 (25.0)
> 50	12 (75.0)
**Location**	
Colon	9 (56.2)
Rectal	7 (43.8)
**Clinical Stage**	
II	3(18.7)
III	7(43.8)
IV	6 (37.5)
**T classification**	
T1	2(12.5)
T2	4 (25.0)
T3	6 (37.5)
T4	4(25.0)
**N classification**	
N0	4 (25.0)
N1	7(43.8)
N2	5 (31.2)
**M classification**	
M0	10(62.5)
M1	6 (37.5)
**Pathologic Type**	
Adenocarcinoma	13 (81.2)
Mucinous Adenocarcinoma	3 (18.8)

### Tumor specimens and patient data

181 paraffin-embedded archived samples of CRC were used in immunohistochemistry, including 99 samples with adjacent non-tumorous (ANT) tissues. All these samples were derived from initial surgery without either preoperative chemotherapy or radiotherapy at Sun Yat-Sen Memorial Hospital between 2002 and 2009. TNM staging and clinicopathologic classification were determined according to the National Comprehensive Cancer Network (NCCN) classification. The clinicopathologic information of the 181 CRC samples was described in Table 3. Overall survival (OS) was defined as the interval between the date of surgery and the date of death or the last known follow up. Additionally, six pairs of matched tumor samples and adjacent non-cancerous tissues (collected immediately after surgery and stored at −80°C were used for real-time PCR analysis.

**Table 3:** Clinicopathological characteristics and DYRK2 expression of 181 patient samples of CRC.

	Number of cases (%)
**Gender**	
Male	122 (67.4)
Female	59 (32.6)
**Age (years)**	
≤ 50	64 (35.4)
> 50	117 (64.6)
**Location**	
Colon	82 (45.3)
Rectal	99 (54.7)
**Clinical Stage**	
I	35 (19.3)
II	41 (22.7)
III	48 (26.5)
IV	57 (31.5)
**T classification**	
T1	14 (7.7)
T2	30 (16.6)
T3	86 (47.5)
T4	51 (28.2)
**N classification**	
N0	85 (47.0)
N1	55 (30.4)
N2	41 (22.7)
**M classification**	
M0	124 (68.5)
M1	57 (31.5)
**Pathologic Differentiation**	
Poor	38 (21.0)
Moderate	129 (71.3)
Well	14 (7.7)
**Expression of DYRK2**	
Low expression	94 (51.9)
High expression	87 (48.1)

### Cell culture and RNA interference

Human colon cancer cell lines HCT116 and SW480 (purchased from ATCC) were maintained in Dulbecco’s modified Eagle’s medium (DMEM, Invitrogen) supplemented with 10% fetal bovine serum (HyClone), 1 mM glutamine and 100 U/ml each of penicillin and streptomycin.The sequence of the DYRK2 siRNA has been reported [10, 27]. The sequences of oligonucleotides1, 2, 3, 4 and 5 targeting DYRK2 mRNA, are Si#1: GGUGCUAUCACAUCUAUAU; Si#2: GGACAGTGCTCACGACACAACCAAA; Si#3: CCACGATCACGTGGCTTACAGGTAT; Si#4: GCCAGGTATGGCATGCCCATTGATA; Si#5: GGGTAGAAGCGGTATTAAA, respectively. These siRNAs were synthesized by GenePharma. Approximately 2 × 10^5^ cells per well were seeded in a 6-well tissue culture dish on the day before transfection. Transfection of 50 nmol siRNAs was performed according to the manufacturer’s instructions by using the LipofectamineTM RNAiMAX transfection reagent (Invitrogen).

### Western blotting and antibodies

Western blotting was performed as described previously [20]. Briefly, cells were lysed in MCLB, and clarified lysates were resolved by SDS-PAGE and transferred to PVDF membranes for western blotting by using ECL detection reagents (Beyotime Co. Haimen, Jiangsu, China). Antibodies against E-cadherin (#3195), N-cadherin (#13116) and Vimentin (5741) were from Cell Signaling Technology. Antibodies against DYRK2 (SC-130743), Fibronectin (sc-9068) and GAPDH (SC-25778) were from Santa Cruz Biotechnology.

### Cell cycle analysis

Cells were harvested, washed with PBS, and fixed with ice-cold 70% ethanol overnight. Cells were then washed in PBS and treated for 20 min at 37°C with RNase A (200 lg/ml), followed by incubation with propidium iodide (25 lg/ml), and analyzed by flow cytometry by using a Cytomics FC 500 flow cytometer (Beckman). The data were analyzed by using Multicycle AV for Windows (Beckman).

### Wound-healing and transwell assays

Cells were trypsinized and seeded equally into 6-well plates to grow to almost full confluence in 24 hours, followed by nonserum starvation for another 24 hours. The cell monolayer was then scratched with a sterile 100 ml pipette tip. After scratching, the cells were washed with PBS and then cultured with a serum-free medium. Cell migration images were captured at time points of 0 h, 24 h and 48 h by an inverted microscope (100X).

For the transwell migration assay, 1.5×105 cells in 200 μl of serum-free DMEM were added to the cell culture inserts with an 8-μm microporous filter without extracellular matrix coating (Becton Dickinson Labware, Bedford, MA). The DMEM medium containing 10% FBS was added to the bottom chamber. After 36 hours of incubation, the cells in the lower surface of the filter were fixed and stained prior to microscopic examination. The number of migrated cells in three random optical fields (100 X) for each filter from triplicate filters was averaged. For the invasion assay, the inserts of the chambers to which the cells were seeded were coated with Matrigel (Becton Dickinson Labware, Bedford, MA). The number of invading cells in three random optical fields (100X) for each filter from triplicate inserts was averaged.

### Immunohistochemistry (IHC) and scoring

Immunohistochemistry was used to study the altered protein expression of DYRK2 in 181 CRC tissues. The procedure was described previously [26]. Briefly, paraffin-embedded tissue blocks were cut at 4 μm thickness before staining. The tissue sections were then deparaffinized at 60°C for 2 hours and rehydrated by two and three changes of xylene and ethanol, respectively. Endogenous peroxidase activity was quenched by incubating with 3% hydrogen peroxide for 30 minutes at room temperature after deparaffinization. Then, antigen retrieval was performed at 121°C for 10 minutes in an autoclave with citrate buffer. Nonspecific binding was blocked by incubating sections with 10% goat serum for 30 minutes at room temperature. The sections were then incubated with a rabbit anti-DYRK2 antibody (1:100; Santa Cruz) overnight at 4°C. After washinged, tissue sections were incubated with biotinylated anti-rabbit secondary antibody for 20 minutes, followed by further incubation with streptavidin-horseradish peroxidase complex. Finally, the sections were developed with diaminobenzidine tetrahydrochloride (DAB) and further counterstained with hematoxylin. The degree of the immunostaining sections was determined by two researchers. One score for the percent of positive cells was set as ≤ 10% = 0, >10% to ≤ 25% = 1, >25% to ≤ 50% = 2, >50% to ≤ 75% = 3, >75% = 4. Another score for the intensity of cytoplasm staining was set as negative = 0, weak = 1, moderate = 2, or strong = 3. The two scores above were then calculated by multiplying as the final score. Low DYRK2 expression was defined as a final score of ≤ 4 and ≥ 6 as a high DYRK2 expression.

### Statistical analysis

All statistical analysis was carried out by using the SPSS 16.0 statistical software package (SPSS Inc., Chicago, IL). Difference of DYRK2 expression between tumor tissues and matched ANT tissues was analyzed by the Wilcoxon signed rank test. Correlation between DYRK2 expression and clinical parameters was analyzed by using the Pearson’s chi-squared test. Survival curves were plotted by the Kaplan-Meier method and compared by the log-rank test. Survival data were evaluated by using univariate and multivariate Cox regression analyses. A two-sided P-value of less than 0.05 was considered to be statistically significant.

## Results

### DYRK2 expression analysis of Oncomine database

**Fig 1:**
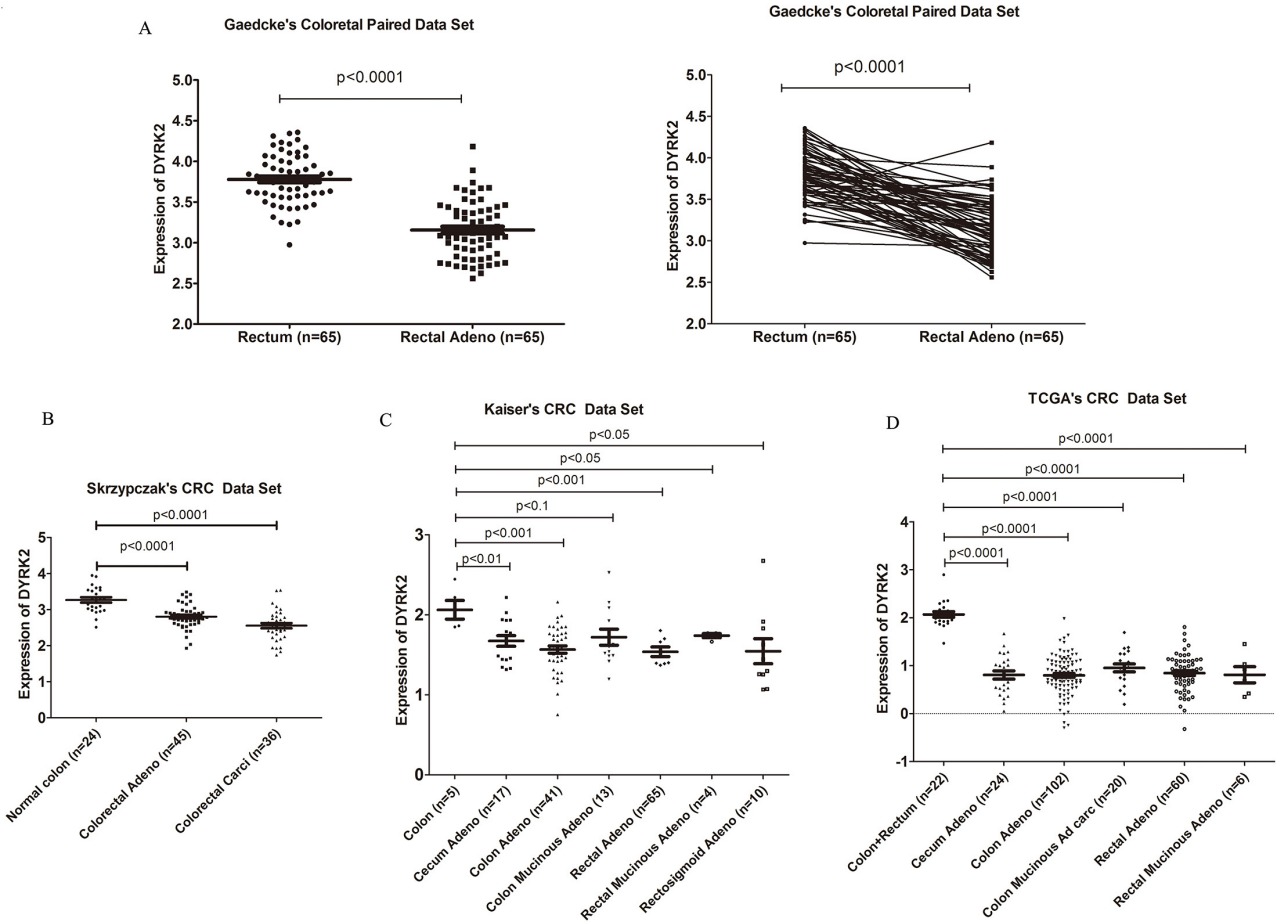
mRNA expression of DYRK2 is lower in CRC compared to the normal colon/rectum. A. Gaedcke's colorectal carcinoma data set: paired normal colon vs paired CRC; B. Skrzypczak's colorectal carcinoma data set: Normal colon vs colorectal adenocarcinoma and colorectal carcinoma; C. Kaiser's colorectal carcinoma data set: normal colon vs different types of CRC; D. TCGA colorectal carcinoma data set: normal colon and rectum vs different type of CRC.

**Fig 2:**
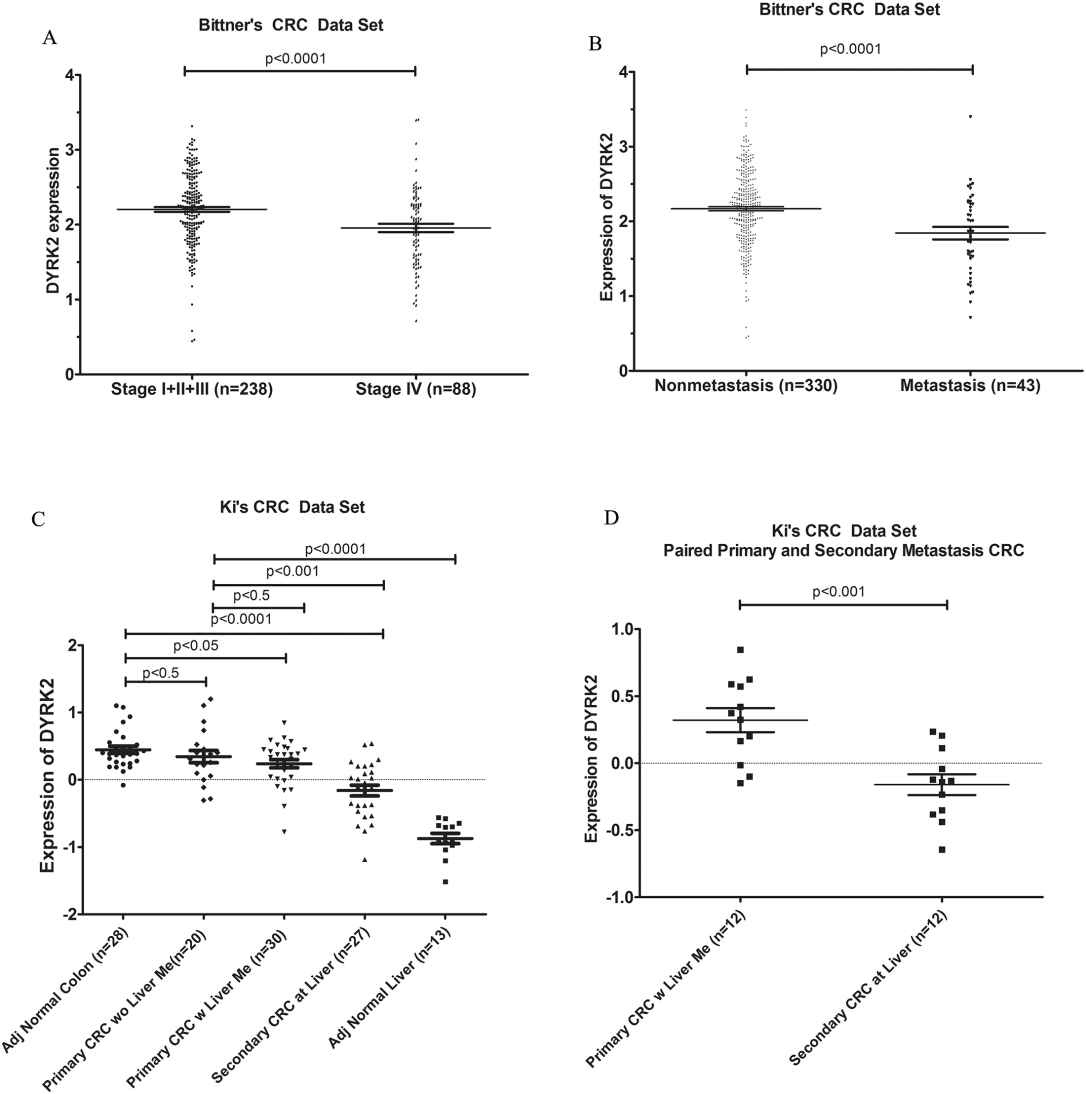
mRNA expression of DYRK2 is lower in high grade and metastatic CRC compared to low grade and non-metastatic CRC. A. Bittner's colorectal carcinoma data set: low grade CRC vs high grade CRC; B. Bittner's colorectal carcinoma data set: non-metastatic CRC vs metastatic CRC; C. Ki's colorectal carcinoma data set: adjacent normal colon vs primary CRC without liver metastatic CRC vs primary CRC with liver metastatic CRC and secondary CRC metastasis at liver; D. Ki's colorectal carcinoma data set: paired primary CRC with liver metastatic CRC vs paired secondary CRC metastasis at liver.

The mRNA levels of kinase from Oncomine databases were analyzed. In CRC, DYRK2 level was found lower in primary or metastatic CRC compared with adjacent normal colon tissues or non-metastatic CRC in all 6 colorectal carcinoma data sets respectively. Specifically, In 65 pairs of normal rectum and primary rectal adenocarcinoma, the level of DYRK2 is lower in cancer tissue compared to paired adjacent normal tissues significantly (Fig 1A). Several other data sets also indicated the similar results (Fig 1B, 1C and 1D). Furthermore, we found that the high grade of CRC and metastatic CRC have less expression of DYRK2 compared with low grades and non-metastatic CRC (Fig 2).

### Decreased expression of DYRK2 in colorectal cancer tissues

**Fig 3:**
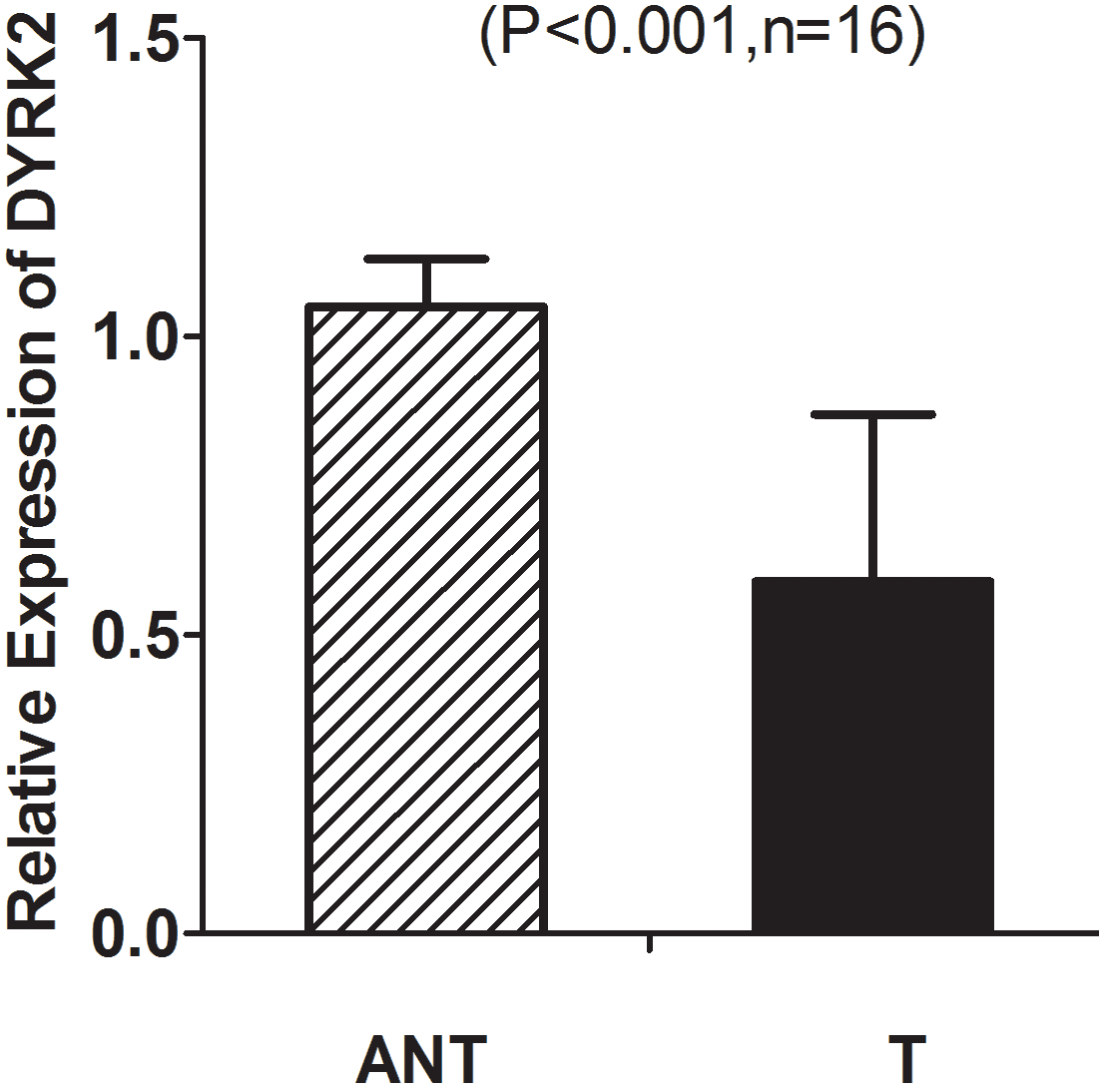
Decreased expression of DYRK2 mRNA in colorectal cancer tissues. Real-time PCR analysis of DYRK2 mRNA expression in sixteen primary colon cancer tissues (T) and adjacent non-cancerous tissues (ANT) paired from the same patient.

**Fig 4:**
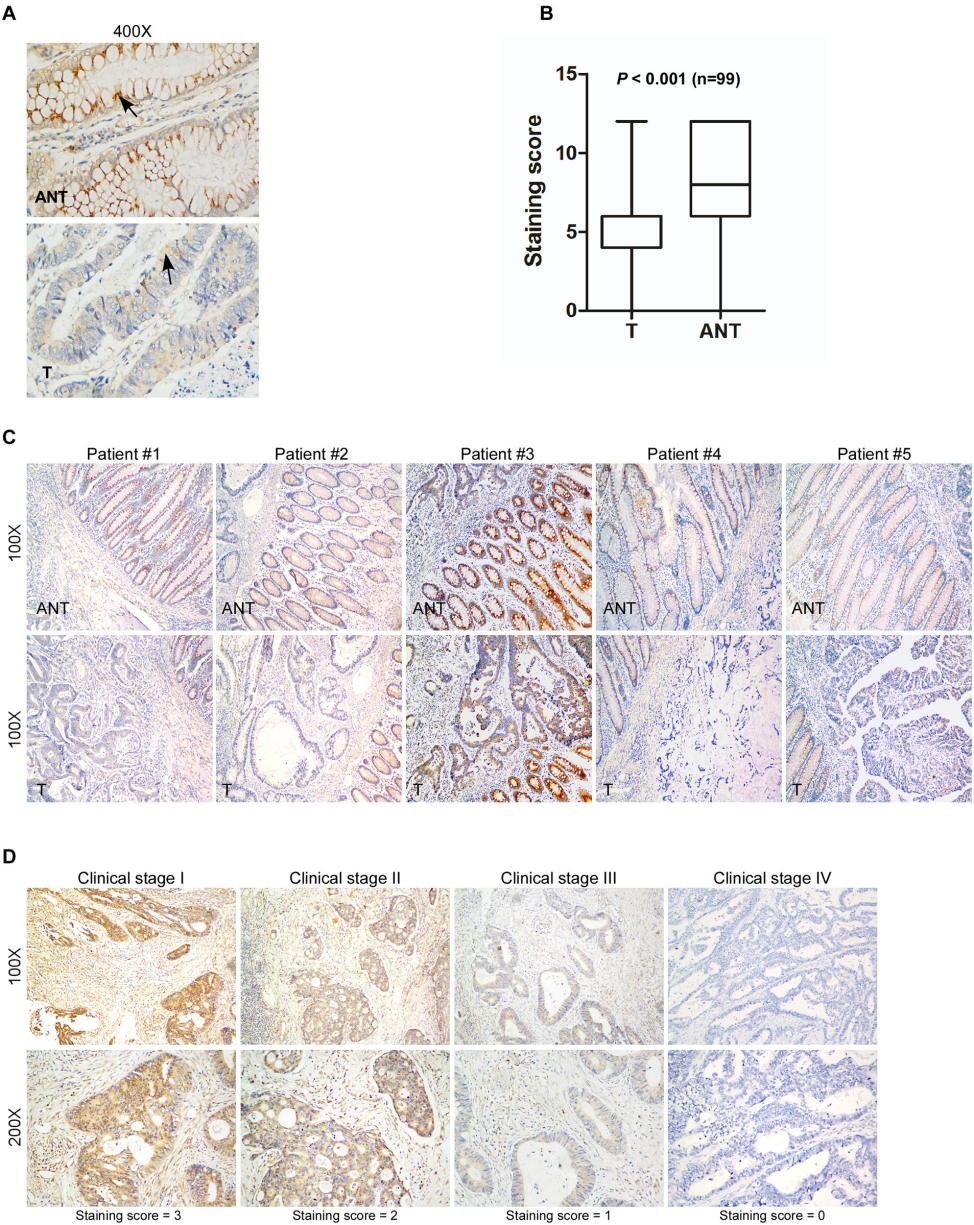
Decreased expression of DYRK2 in paraffin-embedded colorectal cancer tissues and advanced colorectal cancer. A, DYRK2 protein expressed in adjacent non-cancerous tissue (ANT) and colorectal cancer tissue (T) by IHC analysis. B, Quantitative analysis of DYRK2 expression in primary colorectal tumors and adjacent non-cancerous tissues (ANT) by IHC analysis (Wilcoxon signed rank test, n = 99, *P* < 0.001). C, Immuno-staining of DYRK2 in five pairs of representative colorectal tumor tissues (T) with adjacent non-cancerous tissues (ANT). D, Representative IHC analyses of DYRK2 expression at different clinical stages, two magnifications (100X and 200X).

To investigate the role of DYRK2 in tumorigenesis, the expression of DYRK2 in both mRNA and protein levels were evaluated in CRC samples. Decreased expression of DYRK2 mRNA was observed in 16 CRC samples compared with matching ANT samples as observed by real-time PCR (Fig 3). The detail clinicopathologic features listed in the Table 2. We further examined DYRK2 expression by IHC in 181 paraffin-embedded CRC samples, including 99 matched ANT tissues. DYRK2 expression was substantially down regulated in the tumor tissues compared with the ANT tissues (Fig 4B, P < 0.001, Wilcoxon signed rank test), and 5 pairs of the representative slides were shown in Fig 4C, the clinicopathological characteristics of 5 patients were shown in S1 Table.

### DYRK2 inhibits the proliferation, cell migration and invasion of colorectal cancer cells in vitro

**Fig 5:**
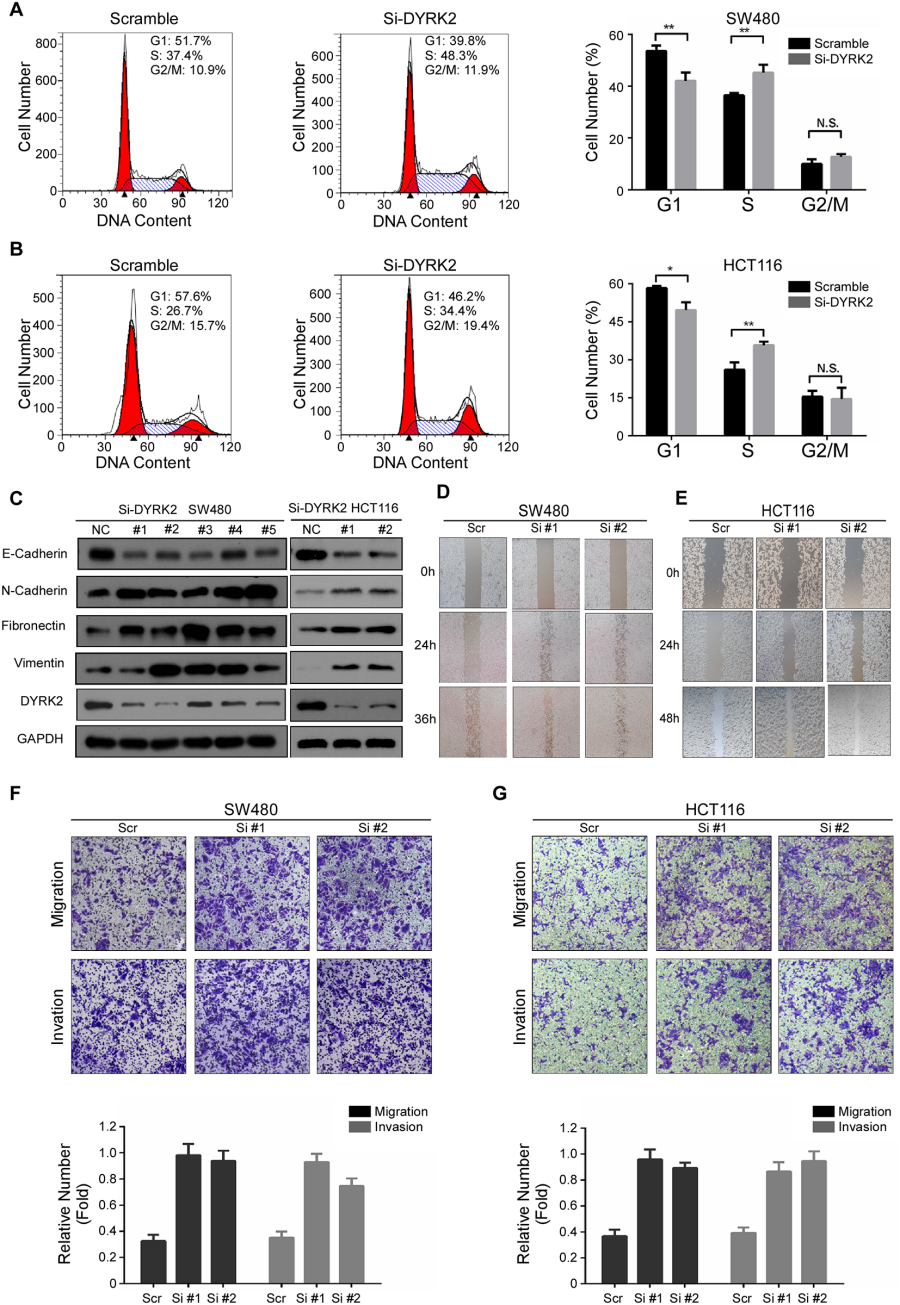
DYRK2 inhibits the proliferation, cell migration and invasion of colorectal cancer cells in vitro. A, B: The effect of DYRK2 depletion on the cell cycle. Experiments were performed in triplicate. C: Western blot analysis of E-cadherin, N-cadherin, Fibronectin and Vimentin expression in the indicated treatment of the CRC cell lines. D, E: The cell's ability of wound gap closure was dramatically enhanced by knocking down DYRK2 both in HCT116 and SW480 cells. F, G: Migratory and invasive abilities of the indicated cell lines *in vitro* was measured by the Transwell assay as described in “Materials and methods”. Bars correspond to mean+ standard error, with a P value calculated by using Student’s *t*-test. *P<0.05, **P<0.001.

To confirm the role of DYRK2 in CRC progression, we further analyzed the effect of DYRK2 depletion on cell cycle progression. The depletion of DYRK2 caused a significant decrease in the G1 population, and yet an increase in the S-phase cell fractions in both HCT116 and SW480 cells when compared with control cells (Fig 5A and 5B). DYRK2 was reported to be not only associated with cancer cell growth but also with cancer metastasis (27, 28). Therefore, we also examined the effect of DYRK2 depletion on CRC metastasis related markers. The western blot assays revealed that the expression of the mesenchymal markers N-cadherin, Vimentin, and Fibronectin, were significantly upregulated, while the epithelial marker E-cadherin was significantly declined upon transient transfection with DYRK2 siRNA (Fig 5C). Furthermore, we evaluated the effect of DYRK2 on CRC cells migration and invasion using wound-healing transwell assays. As shown in Fig 5D and 5E, the cell's ability of wound healing was dramatically enhanced after the depletion of endogenous DYRK2 in HCT116 and SW480 cells. Likewise, the transwell assays showed that the abilities of migration and invasion were also dramatically enhanced by knocking down DYRK2 (Fig 5F and 5G). Taken together, these data confirmed that DYRK2 inhibited cell proliferation, invasion and migration in both HCT116 and SW480 cells and functioned as a tumor suppressor in CRC cells.

### Correlation between DYRK2 expression and clinicopathologic features of CRC

Clinical characteristics of CRC included 35 cases of clinical stage I (19.3%), 41 cases of clinical stage II (22.7%), 48 cases of clinical stage III (26.5%), and 57 cases of clinical stage IV (31.5%) CRC. 87 of total 181 CRC cases (48.1%) highly expressed DYRK2; 94 cases (51.9%) had low expression of DYRK2 (Table 3).

A strong correlation occurred between DYRK2 expression (determined by using IHC staining), and clinicopathologic characteristics of these CRC patients, including tumor site (P = 0.023), clinical stage (P = 0.006), Nodal (N) classification (P = 0.017), Metastasis (M) classification (P = 0.004), and mortality (P < 0.001) (Table 4). In contrast, DYRK2 expression did not correlate with gender, age and tumor differentiations (Table 4). Representative IHC stained slides showed the correlation of DYRK2 expression with clinical stages (Fig 4D).

**Table 4:** Correlation between DYRK2 expression and clinicopathological characteristics of CRC patients.

Characteristics		DYRK2		Chi-square Test P-value
		Low or none	High	
		No. cases (%)	No. cases (%)	
Gender	Female	32 (34.0)	27 (31.0)	0.751
	Male	62 (66.0)	60 (69.0)	
Age (years)	≤ 50	35 (37.2)	29 (33.3)	0.642
	> 50	59 (66.9)	58 (66.7)	
Location	colon	35 (46.9)	47 (45.6)	**0.023**
	rectal	59 (53.1)	40 (54.4)	
Clinical Stage	I	12 (12.8)	23 (26.4)	**0.006**
	II	17 (18.1)	24 (27.6)	
	III	26 (27.6)	22 (25.3)	
	IV	39 (41.5)	18 (20.7)	
T classification	T1+T2	18 (19.1)	26 (29.9)	0.118
	T3+T4	76 (80.9)	61 (70.1)	
N classification	No	36 (38.3)	49 (56.3)	**0.017**
	Yes	58 (61.7)	38 (43.7)	
M classification	M0	55 (58.5)	69 (79.3)	**0.004**
	M1	39 (41.5)	18 (20.7)	
Pathologic Differentiation	Poor	22 (23.4)	16 (18.4)	0.365
	Moderate	67 (71.3)	62 (71.3)	
	Well	5 (5.3)	9 (10.3)	
	Dead	56 (59.6)	21 (24.1)	

### Association between DYRK2 expression and patient survival

**Fig 6:**
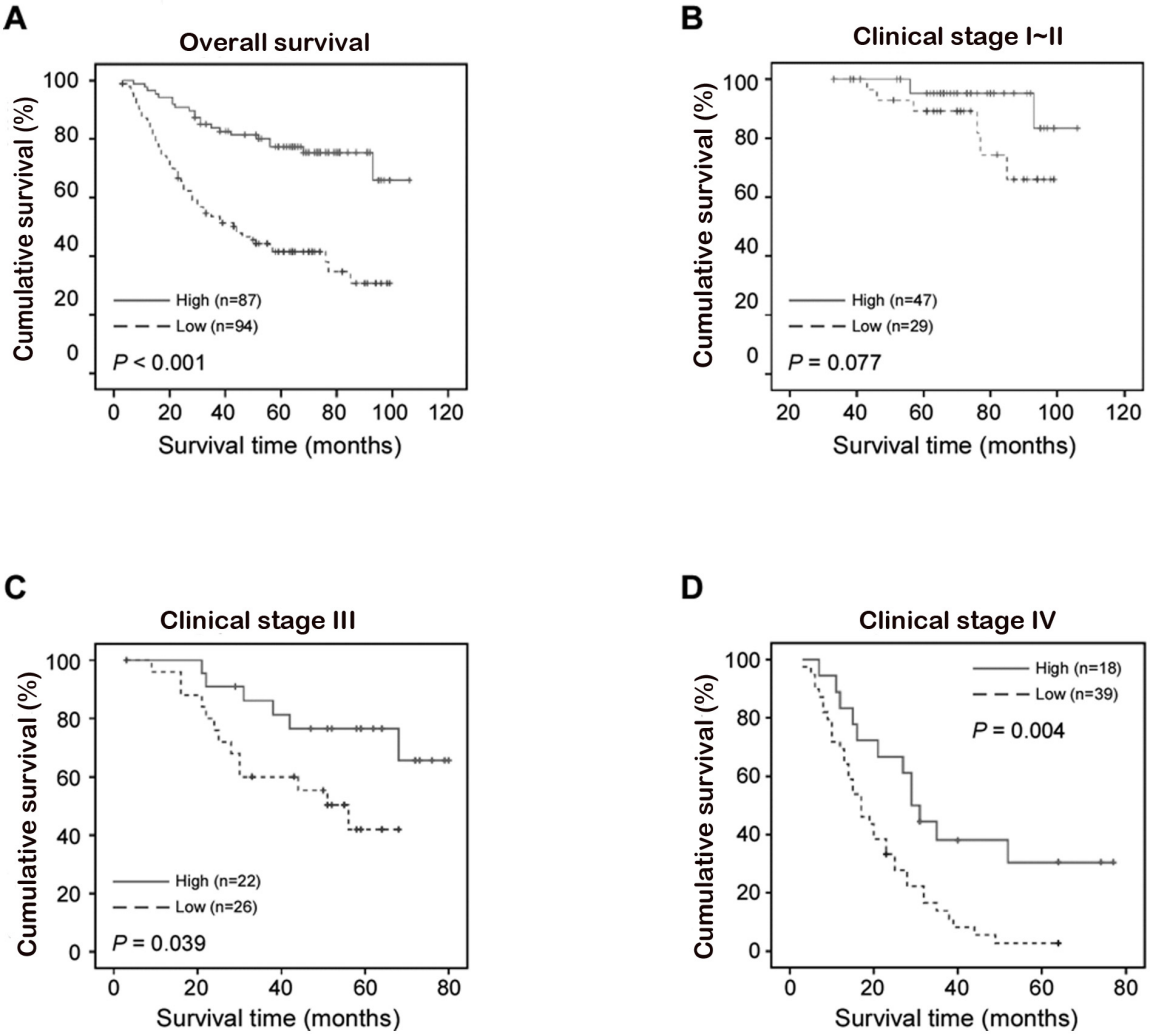
Kaplan-Meier curves with univariate analyses (log-rank) for patients with low DYRK2 expression (dotted line) versus high DYRK2 expression (bold line). A, Overall survival of patients (clinical stages I-IV) with either low or high DYRK2 expression. B, Overall survival of patients (clinical stages I-II) with either low or high DYRK2 expression. C-D, Overall survival of patients (clinical stages III (C) and IV (D)) with either low or high DYRK2 expression.

A clear positive correlation existed between DYRK2 expression and the overall survival of patients. Lower DYRK2 expression correlated with a shorter overall survival time (OS) (median OS: 44.0 months) compared with higher DYRK2 expression (median OS: not reached up to 77.0 months) (Fig 6A, P < 0.001). The overall two-, three-, and five-year accumulative survival rates of patients with higher DYRK2 expression were 81.4%, 77.4%, and 75.4%, respectively. For patients with lower DYRK2 expression, the rates were 77.4%, 65.6%, and 44.3%, respectively (Fig 6A). Furthermore, a significant correlation of DYRK2 expression with the OS was found in the advanced clinical stages (stage III and IV) (Fig 6D, P = 0.039 (stage III); P = 0.004 (stage IV)). Moreover, Cox regression revealed that DYRK2 expression was an independent prognostic factor for CRC patients, as well as N classification, M classification and pathologic differentiation (Table 5).

**Table 5:** Univariate and multivariate analysis of various prognostic parameters in patients with CRC Cox-regression analysis.

Variables	Univariate analysis		Multivariate analysis		
	No.	P value	Hazard Ratio	95% CI	P value
**DYRK2**					
low expression	94	**<0.001**	0.336	0.200–0.564	**<0.001**
high expression	87				
**T classification**					
T1	14	**<0.001**	1.043	0.750–1.452	0.801
T2	30				
T3	86				
T4	51				
**N classification**					
N0	85	**<0.001**	1.609	1.174–2.204	**0.003**
N1	55				
N2	41				
**M classification**					
M0	124	**<0.001**	8.276	4.600–14.888	**<0.001**
M1	57				
**Differentiation**					
Poor	38	0.559	0.63	0.418–0.952	**0.028**
Moderate	129				
Well	14				

## Discussion

In recent years, the incidences of CRC have rapidly increased and become the second leading cause of cancer death in China [27]. Although the clinical treatment of CRC has made great progress, the clinical outcome of advanced CRC is still poor [28]. TNM classification remains the “gold-standard” prognostic measure of this disease [29]. Sadly, disease-free and overall survival data still vary even on the same TNM stage. Apparently, better prognostic indexes are urgently needed to guide therapy and benefit patients.

Protein phosphorylation is a key post-translational modification. In eukaryotes, protein kinases are pivotal enzymes regulating a broad spectrum basic cellular processes by providing a phosphate to substrates [30]. The dual-specificity tyrosine-regulated kinases (DYRKs) belong to the CMGC protein kinases super family and were found to be pleiotropic regulators of cellular functions [3, 31]. DYRK2 behaves as a tumor suppressor in many human cancers. It empowers p53 to induce apoptosis in response to DNA damage via the phosphorylation of Ser 46. Silencing DYRK2 function attenuates (adriamycin) -induced apoptosis [10]. Paradoxically, DYRK2 can induce cell apoptosis in a p53-independent manner [10]. Studies of Taira *et*.*al* suggested that DYRK2 regulated tumor progression *in vivo* an *in vitro* through the modulation of c-Jun and c-Myc. The knockdown of DYRK2 shortened the G1 phase and accelerated cell proliferation due to the escape of c-Jun and c-Myc from ubiquitination-mediated degradation [32]. Besides, the knockdown of DYRK2 promoted Epithelial–mesenchymal transition (EMT) and cancer invasion in human breast cancer cells [33]. In our study, we examined the effect of DYRK2 invasion on CRC and revealed that the same phenomena. While DYRK2 was depleted, the invasion ability of the CRC cell line SW480 and HCT116 were upgraded (Fig 5A, 5B and 5C). Furthermore, DYRK2 on CRC cells’ migration ability of wound healing was dramatically enhanced after the depletion of endogenous DYRK2 in HCT116 and SW480 cells (Fig 5D and 5E). Taken together, these data confirmed that DYRK2 inhibited cell invasion and migration in both HCT116 and SW480 cells and functioned as a tumor suppressor in CRC cells. Moreover, patients with DYRK2-positive tumors in recurrent non-small cell lung cancer has substantially benefited from chemotherapy as compared with those with DYRK2-negative tumors [14]. This is consistent with our results that showed a relative favorable survival in patients with higher expression of DYRK2 in clinical stage IV CRC (Fig 6D).

Our current study presented the first piece of evidence that DYRK2 was down regulated in colon cancer at both transcriptional and translational levels. Real-time PCR by using 16 pairs of human colorectal cancer tissues showed the down-expression of DYRK2 mRNA compared with matching adjacent non-tumorous tissues (Fig 3, P < 0.001, paired t test). Accordingly, IHC analysis showed down expression of DYRK2 protein in 99 CRC tissues compared with matching adjacent non-tumorous tissues (Fig 4B, P < 0.001, Wilcoxon signed rank test). Lower protein levels of DYRK2 significantly correlated with tumor location in rectum, advanced tumor stage and unfavorable prognosis in clinical stage III and IV CRC patients. Notably, the prognostic value of tumor site in CRC remains controversial. Some studies revealed that survival of rectal cancer was worse than colon cancer [34], while others found the opposite [35]. Leicester et al reported that CRC and normal childhood colorectal samples have similar behavior in increased proliferation and decreased apoptosis with a significantly lower expression of DYRK2 compared to healthy colonic mucosa from adults [36]. In our study, all samples are from adult patients. Interestingly, our current study observed more cases of decreased expression of DYRK2 in rectal cancer than in colon cancer (Table 4, P = 0.023). Remarkably, multivariate analysis implied DYRK2 as an independent prognostic factor of survival in CRC patients.

## Conclusions

In conclusion, our study revealed that the dual-specificity tyrosine-phosphorylation-regulated kinase DYRK2 behaves as a tumor suppressor in the CRC Function tests confirmed that DYRK2 inhibited cell proliferation, invasion and migration in both HCT116 and SW480 cells and functioned as a tumor suppressor in CRC cells. The assessment of DYRK2 expression by IHC at diagnosis may help guide therapeutic decisions. DYRK2 expression was highly associated with clinical stages, N classification and an M classification of CRC. Patients with reduced DYRK2 expression had a shorter OS, and acted as an independent prognostic factor.

## Supporting Information

S1 Table Clinicopathological characteristics and DYRK2 expression of 5 patient samples of Fig 4C. (DOCX)

## Abbreviations

DYRKs: Dual-specificity tyrosine-phosphorylation-regulated kinases
DYRK2: Dual-specificity tyrosine-phosphorylation-regulated kinase 2
CRC: Colorectal cancer
NCCN: National Comprehensive Cancer Network
DAB: Diaminobenzidine tetrahydrochloride
IHC: Immunohistochemistry
ANT: Adjacent non-tumorous tissues
OS: Overall survival
GSK3β: Glycogen synthase kinase 3β
MSI: Microsatellite instability
