# Supplementary material for: Low Expression of DYRK2 (Dual Specificity Tyrosine Phosphorylation Regulated Kinase 2) Correlates with Poor Prognosis in Colorectal Cancer
Source: PLoS One. 2016 Aug 17;11(8):e0159954. doi: 10.1371/journal.pone.0159954 (PMC4988784; doi:10.1371/journal.pone.0159954)
Supplement: S1 Table — (DOCX) [file pone.0159954.s001.docx]

Supplymental Table 1. Clinicopathological characteristics and DYRK2 expression of 5 patient samples of Figure4c

| Patients | Age | Gender | Pathology ID | Tumor  location | TNM phase | Clinic stage | Differentiation dgree | Pathological diagnosis |
| --- | --- | --- | --- | --- | --- | --- | --- | --- |
| 1 | 49 | Male | 418500 | Colon | T3N1M1 | IV | Moderate | Adenocarcinoma |
| 2 | 28 | Male | 367295 | Colon | T4N1M0 | III | Moderate | Adenocarcinoma |
| 3 | 47 | Female | 313710 | Colon | T2N0M0 | I | Moderate | Adenocarcinoma |
| 4 | 60 | Male | 347139 | Rectal | T4N1M0 | III | Poor | Mucinous adenocarcinoma |
| 5 | 58 | Male | 314970 | Colon | T2N0M0 | I | Poor | Adenocarcinoma |
